# *In Vitro* and *in Vivo* Metabolism of Verproside in Rats

**DOI:** 10.3390/molecules171011990

**Published:** 2012-10-12

**Authors:** Min Gi Kim, Deok-Kyu Hwang, Hyeon-Uk Jeong, Hye Young Ji, Sei-Ryang Oh, Yongnam Lee, Ji Seok Yoo, Dae Hee Shin, Hye Suk Lee

**Affiliations:** 1 Drug Metabolism & Bioanalysis Laboratory, College of Pharmacy, The Catholic University of Korea, Bucheon 420-743, Korea; Email: minki8637@naver.com (M.G.M.); myhdg@naver.com (D.-K.H.); wjd1375@hanmail.net (H.-U.J.); hychi@catholic.ac.kr (H.Y.J.); 2 Natural Medicine Research Center, Korea Research Institute of Bioscience and Biotechnology, Ochang-eup, Cheongwon-gun, Chungbuk 363-883, Korea; Email: seiryang@kribb.re.kr; 3 Central R&D Institute, Yungjin Pharm. Co., Ltd., Suwon 443-270, Korea; Email: nami0209@yungjin.co.kr (Y.L.); jsyoo@yungjin.co.kr (J.S.Y.); jkyk58@yungjin.co.kr (D.H.S.)

**Keywords:** verproside metabolism, rat bile, rat urine, rat hepatocytes, LC-HRMS

## Abstract

Verproside, a catalpol derivative iridoid glycoside isolated from *Pseudolysimachion rotundum *var*. subintegrum*, is a biologically active compound with anti-inflammatory, antinociceptic, antioxidant, and anti-asthmatic properties. Twenty-one metabolites were identified in bile and urine samples obtained after intravenous administration of verproside in rats using liquid chromatography-quadrupole Orbitrap mass spectrometry. Verproside was metabolized by *O*-methylation, glucuronidation, sulfation, and hydrolysis to verproside glucuronides (M1 and M2), verproside sulfates (M3 and M4), picroside II (M5), M5 glucuronide (M7), M5 sulfate (M9), isovanilloylcatalpol (M6), M6 glucuronide (M8), M6 sulfate (M10), 3,4-dihydroxybenzoic acid (M11), M11 glucuronide (M12), M11 sulfates (M13 and M14), 3-methyoxy-4-hydroxybenzoic acid (M15), M15 glucuronides (M17 and M18), M15 sulfate (M20), 3-hydroxy-4-methoxybenzoic acid (M16), M16 glucuronide (M19), and M16 sulfate (M21). Incubation of verproside with rat hepatocytes resulted in thirteen metabolites (M1–M11, M13, and M14). Verproside sulfate, M4 was a major metabolite in rat hepatocytes. After intravenous administration of verproside, the drug was recovered in bile (0.77% of dose) and urine (4.48% of dose), and *O*-methylation of verproside to picroside II (M5) and isovanilloylcatalpol (M6) followed by glucuronidation and sulfation was identified as major metabolic pathways compared to glucuronidation and sulfation of verproside in rats.

## 1. Introduction

Verproside, a catalpol derivative iridoid glycoside isolated from *Pseudolysimachion spurium*, *Pseudolysimachion rotundum *var*. subintegrum*, *Pseudolysimachion longifolium* and *Veronica anagallis-aquatica etc*., shows potent anti-inflammatory, antioxidant, and antinociceptic activities [1,2,3,4,5]. Verproside also significantly reduced the levels of total immunoglobulin E, interleukin-4 and interleukin-13 in the plasma and bronchoalveolar lavage fluid of an ovalbumin-induced asthmatic mouse model, suggesting that verproside is a strong candidate as an anti-asthmatic drug [6].

The pharmacokinetics of verproside were evaluated in rats after intravenous (dose range 2–10 mg/kg) and oral (dose range 20–100 mg/kg) administration [7,8]. Short half-life (12.2–16.6 min), high systemic clearance (56.7–86.2 mL/min/kg), and low renal clearance (2.7–4.1 mL/min/kg) of verproside after intravenous administration and very low oral bioavailability of verproside (less than 0.5%) in male Sprague-Dawley rats suggest that verproside may be extensively metabolized [8]. Isovanilloylcatalpol was identified as a metabolite of verproside after intravenous administration, but was not detected after oral administration to rats in our previous study [8]. Therefore, it is necessary to elucidate the other metabolic pathways of verproside in rats, aside from the formation of isovanilloylcatalpol, for characterization of its pharmacodynamics and toxicity.

In this work the *in vivo* metabolites of verproside in the bile and urine samples obtained after intravenous administration of verproside, and the predominant metabolites formed from *in vitro* incubation of verproside and its possible metabolites from rat liver S9 fractions and hepatocytes were elucidated using liquid chromatography-high resolution quadrupole Orbitrap mass spectrometry (LC-HRMS).

## 2. Results and Discussion

**Figure 1:**
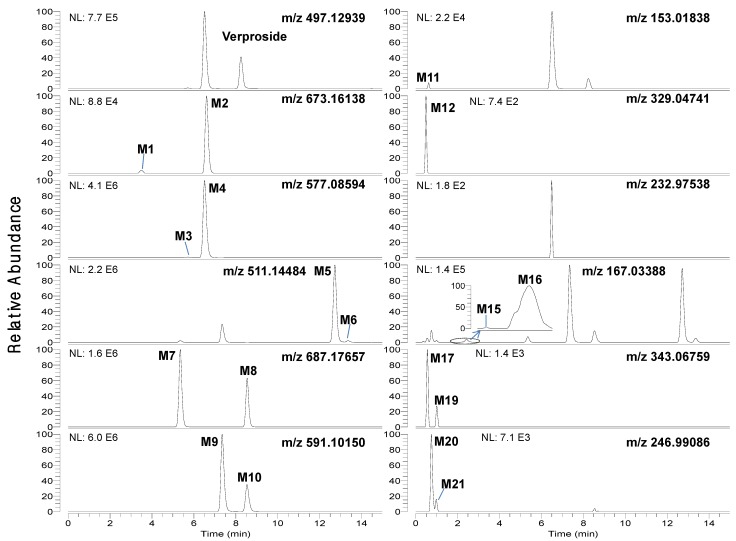
Extracted ion chromatograms of verproside and its possible metabolites in the bile samples obtained for 6 hours after intravenous administration of verproside at a dose of 10 mg/kg to a Sprague Dawley rat using 5 ppm mass accuracy.

**Figure 2:**
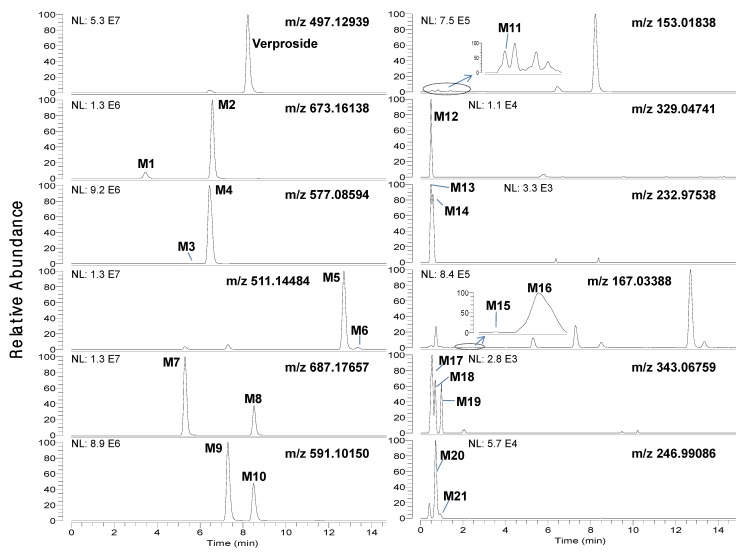
Extracted ion chromatograms of verproside and its possible metabolites in the urine samples obtained for 6 hours after intravenous administration of verproside at a dose of 10 mg/kg to a Sprague Dawley rat.

LC-HRMS analysis of the bile and urine samples obtained after intravenous injection of verproside to rats resulted in twenty-one metabolites (M1–M21), along with unchanged verproside (Figure 1 and Figure 2). The accurate mass of deprotonated molecular ions ([M−H]^−^) and the retention times (t_R_) for verproside and its twenty-one metabolites, M1-M21, are shown in Table 1. Incubation of verproside with rat hepatocytes resulted in thirteen metabolites, namely M1–M11, M13, and M14.

Chromatographic separation of the metabolites was needed to unambiguously identify the structure of the metabolites since some metabolites were identified to have the same [M−H]^−^ ions as follows: *m/z* 511.14484 for M5 (t_R_, 12.73 min) and M6 (t_R_, 13.37 min), *m/z* 687.17657 for M7 (t_R_, 5.35 min) and M8 (t_R_, 8.55 min), *m/z* 591.10150 for M9 (t_R_, 7.33 min) and M10 (t_R_, 8.52 min), *m/z* 167.03388 for M15 (t_R_, 1.90 min) and M16 (t_R_, 2.30 min), *m/z* 343.06759 for M17 (t_R_, 0.69 min), M18 (t_R_, 0.86 min) and M19 (t_R_, 0.96 min), and *m/z* 246.99086 for M20 (t_R_, 0.90 min) and M21 (t_R_, 1.24 min) (Figure 1 and Figure 2, Table 1). After many trials with different columns and mobile phase combinations, verproside and its twenty-one metabolites were well separated on a Halo C18 column using a gradient elution of methanol and 1 mM ammonium formate (pH 3.1).

**Figure 3:**
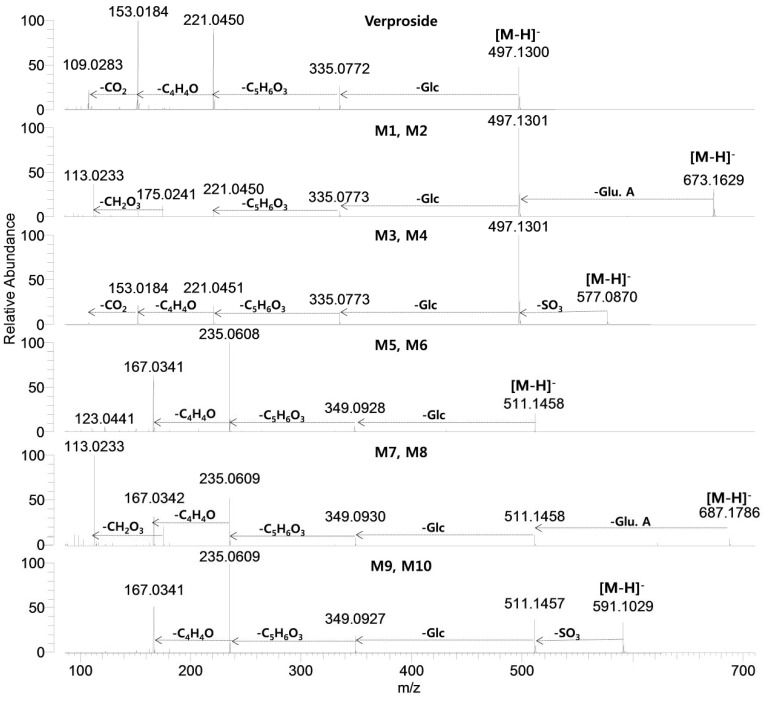
Product scan spectra of verproside and its metabolites (M1–M10) obtained by LC-HRMS analysis of the rat urine and bile samples obtained after intravenous administration of verproside at a dose of 10 mg/kg to a Sprague Dawley rat. Glc: glucose; Glu.A: glucuronosyl.

**Figure 4:**
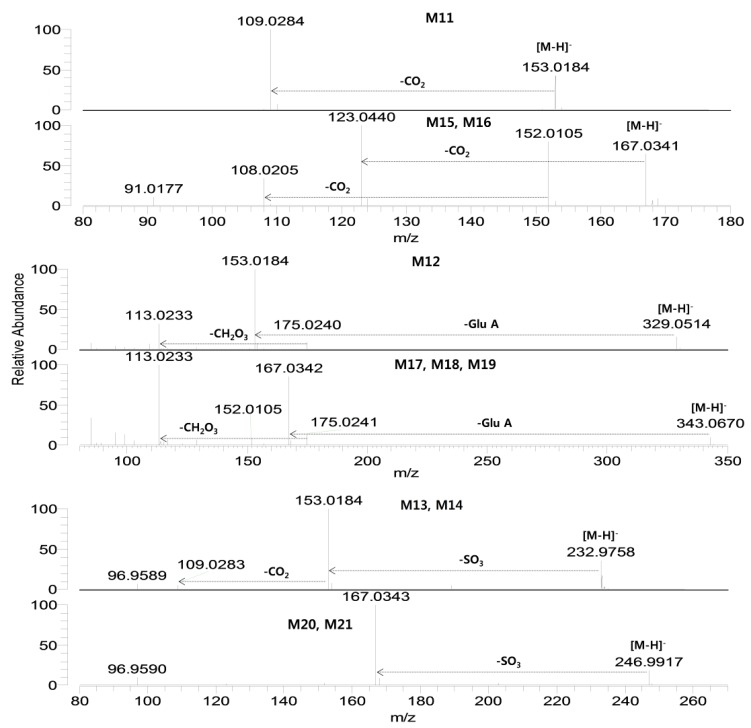
Product scan spectra of verproside and its metabolites (M11–M21) obtained by LC-HRMS analysis of the rat urine and bile samples obtained after intravenous administration of verproside at a dose of 10 mg/kg to a Sprague Dawley rat. Glc: glucose; Glu.A: glucuronosyl.

The identification of twenty-one metabolite peaks was achieved using the accurate mass and the prominent and informative product ions from the product scan spectra (Table 1, Figure 3 and Figure 4). The mass deviation between experimental and theoretical *m/z* ratio for each metabolite was less than 5 ppm, indicating good correlation between the theoretical mass calculated from the molecular elemental composition and the experimental mass obtained after full scan MS analysis. The identity of some metabolites of verproside in the bile and urine samples was ascertained by comparing their retention times and accurate masses with those of the authentic standards. Because no authentic standards were available for the metabolites of sulfates and glucuronides, sulfates and glucuronides were also identified by treating the bile and urine samples with b-glucuronidase or sulfatase and by *in vitro* metabolism of verproside and its authentic metabolite standards including isovanilloylcatalpol, picroside II, 3,4-dihydroxybenzoic acid, 3-methoxy-4-hydroxybenzoic acid and 4-methoxy-3-hydroxy-benzoic acid with rat liver S9 fractions in the presence of uridine 5'-diphosphoglucuronic acid (UDPGA) and 3'-phosphoadenosine-5'-phosphosulfate (PAPS), respectively.

The product scan spectra of verproside yielding an [M−H]^−^ ion at *m/z* 497.12939 generated characteristic product ions at *m/z* 335.0772 (loss of glucose from the [M−H]^−^ ion), *m/z* 221.0450 (loss of C_5_H_6_O_3_ due to the breakdown of the iridoid moiety from *m/z* 335.0772) [9], *m/z* 153.0184 (3,4-dihydroxybenzoyl moiety) and *m/z* 109.0284 (loss of carboxyl group from *m/z* 153.0184) (Figure 3). 

M1 and M2 each produced a [M−H]^−^ ion at *m/z* 673.16138, which is 176 amu higher than the [M−H]^−^ ion of verproside, indicating glucuronidation of verproside. M1 and M2 yielded characteristic product ions at *m/z* 497.1302 (loss of the glucuronosyl moiety from [M−H]^−^ ion), *m/z* 335.0775, *m/z* 221. (loss of C_5_H_6_O_3_ from *m/z* 335.0775), *m/z* 175.0241 (glucuronosyl moiety), and *m/z* 113.0233 (loss of CO_2_ and H_2_O from *m/z* 175.0241) (Figure 3). M2 was identified as the major metabolite after incubation of verproside with rat liver S9 fraction in the presence of UDPGA and rat hepatocytes. Treatment of the bile and urine samples with b-glucuronidase resulted in the increase of the peak area for verproside and the disappearance of M1 and M2 peaks. From these results, M1 and M2 were tentatively identified as verproside glucuronides, but the exact site for glucuronidation could not be determined. The amount of M2 was more than M1 in the bile and urine samples.

**Table 1:** Accurate mass and retention time of verproside and its metabolites identified in the urine and bile samples obtained after intravenous injection of verproside at a dose of 10 mg/kg in male Sprague Dawley rats.

Metabolite	Compound name	Formula	Detected accurate mass ( *m/z*)	Error (ppm)	Retention time (min)
	Verproside	C_22_H_26_O_13_	497.12939	−1.5	8.28
M1, M2	Verproside glucuronide	C_28_H_34_O_19_	673.16138	−0.9	3.50, 6.62
M3, M4	Verproside sulfate	C_22_H_26_SO_16_	577.08594	−1.6	5.68, 6.50
M5	Picroside II	C_23_H_28_O_13_	511.14484	−1.7	12.73
M6	Isovanilloylcatalpol	C_23_H_28_O_13_	511.14484	−1.7	13.37
M7	Picroside II glucuronide	C_29_H_36_O_19_	687.17657	−1.7	5.35
M8	Isovanilloylcatalpol glucuronide	C_29_H_36_O_19_	687.17657	−1.7	8.55
M9	Picroside II sulfate	C_23_H_28_SO_16_	591.10150	−1.5	7.33
M10	Isovanilloylcatalpol sulfate	C_23_H_28_SO_16_	591.10150	−1.5	8.52
M11	3,4-Dihydroxybenzoic acid	C_7_H_6_O_4_	153.01838	−0.6	0.72
M12	3,4-Dihydroxybenzoic acid glucuronide	C_13_H_14_O_10_	329.04741	3.5	0.58
M13, M14	3,4-Dihydroxybenzoic acid sulfate	C_7_H_6_SO_7_	232.97538	3.0	0.51, 0.60
M15	3-Methoxy-4-hydroxybenzoic acid	C_8_H_8_O_4_	167.03388	−0.2	1.90
M16	3-Hydroxy-4-methoxybenzoic acid	C_8_H_8_O_4_	167.03388	0.2	2.30
M17, M18	3-Methoxy-4-hydroxybenzoic acid glucuronide	C_14_H_16_O_10_	343.06759	3.6	0.53, 0.69
M19	3-Hydroxy-4-methoxybenzoic acid glucuronide	C_14_H_16_O_10_	343.06759	3.8	0.96
M20	3-Methoxy-4-hydroxybenzoic acid sulfate	C_8_H_8_SO_7_	246.99086	3.3	0.90
M21	3-Hydroxy-4-methoxybenzoic acid sulfate	C_8_H_8_SO_7_	246.99086	3.4	1.24

M3 and M4 each showed an [M−H]^−^ ion at *m/z* 577.08594, which is 80 amu higher than the [M−H]^−^ ion of verproside, suggesting sulfation of verproside. The product scan spectra of M3 and M4 generated characteristic product ions at *m/z* 497.1301 (loss of SO_3_ from [M−H]^−^ ion), *m/z* 335.0773, *m/z* 221.0451 and *m/z* 153.0184 (Figure 3). M3 and M4 were produced after incubation of verproside with rat liver S9 fraction in the presence of PAPS and with rat hepatocytes. After sulfatase treatment of the bile and urine samples, the peak area of verproside was increased but M3 and M4 peaks were decreased. These results indicate that M3 and M4 might be verproside sulfates, but the position of sulfation was not accurately identified. M4 was identified as a major metabolite after incubation of verproside in rat hepatocytes, and the amount of M4 was more than that of M3 in the bile and urine samples.

M5 and M6 each showed a [M−H]^−^ ion at *m/z* 511.14484, which is 14 amu more than the [M−H]^−^ ion of verproside, suggesting that those metabolites were formed from verproside via *O*-methylation. By comparison with the retention times and the product scan spectra of corresponding authentic standards, M5 and M6 were identified as picroside II and isovanilloylcatalpol, respectively. M5 and M6 produced characteristic product ions at *m/z* 349.0928 (loss of glucose from [M−H]^−^ ion), *m/z* 235.0608 (loss of C_5_H_6_O_3_ due to the breakdown of the iridoid moiety from *m/z* 349.0928), *m/z* 167.0341 (3-hydroxy-4-methoxybenzoyl or 3-methoxy-4-hydroxybenzoyl moiety), and *m/z* 123.0441 (loss of CO_2_ from *m/z* 167.0341) (Figure 3). Because cathechol *O*-methyltransferase favors 3-*O*-methylation over 4-*O*-methylation, the amount of picroside II (M5) was more than that of isovanilloylcatalpol (M6) in the bile and urine samples. 

M7 and M8 each showed a [M−H]^−^ ion at *m/z* 687.17567, which is 176 amu higher than the [M−H]^−^ ion of M5 (picroside II) and M6 (isovanilloylcatalpol), indicating glucuronidation of M5 and M6. M7 and M8 generated characteristic product ions at *m/z* 511.1458 (loss of the glucuronosyl moiety from the [M−H]^−^ ion), *m/z* 349.0930 (loss of glucuronic acid and glucose from the [M−H]^−^ ion), *m/z* 235.0609, *m/z* 167.0341, *m/z* 175.0240 (glucuronosyl moiety) and *m/z* 113.0233 (loss of CO_2_ and H_2_O from *m/z* 175.0240) (Figure 3). While M7 was produced after incubation of picroside II (M5) with rat liver S9 fraction in the presence of UDPGA, M8 was produced from isovanilloylcatalpol. b-glucuronidase treatment of the bile and urine samples increased the peak areas of M5 (picroside II) and M6 (isovanilloylcatalpol), but M7 and M8 peaks were not detected. These results indicate that M7 and M8 may be M5 (picroside II) glucuronide and M6 (isovanilloylcatalpol) glucuronide, respectively, but the position of glucuronidation was not accurately identified. These results support the results of Li *et al.* [10] that identified picroside II glucuronide in the bile samples obtained after intravenous administration of picroside II in rats.

M9 and M10 each showed a [M−H]^−^ ion at *m/z* 591.10150, which was 80 amu higher than the [M−H]^−^ ion of M5 (picroside II) and M6 (isovanilloylcatalpol), suggesting sulfation of M5 and M6. Incubation of picroside II and isovanilloylcatalpol with rat liver S9 fraction in the presence of PAPS resulted in the formation of M9 and M10, respectively. M9 and M10 were identified after incubation of verproside with rat hepatocytes. The product scan spectra of M9 and M10 generated characteristic product ions at *m/z* 511.1457 (loss of SO_3_ from the [M−H]^−^ ion), *m/z* 349.0927 (loss of glucose from *m/z* 511.1463), *m/z* 235.0609, and *m/z* 167.0341. Sulfatase treatment of the bile and urine samples increased the peak areas of M5 (picroside II) and M6 (isovanilloylcatalpol), but decreased the M9 and M10 peaks. These results indicate that M9 and M10 might be M5 (picroside II) sulfate and M6 (isovanilloylcatapol) sulfate, respectively, supporting the result of Li *et al.* [10] that showed picroside II was metabolized to picroside II sulfate as well as picroside II glucuronide in rats.

M11 showed a [M−H]^−^ ion at *m/z* 153.01838 and was identified as 3,4-dihydroxybenzoic acid by comparison with the accurate mass, retention time, and product scan spectrum of the corresponding authentic standard (Figure 4). Incubation of verproside with rat hepatocytes resulted in M11.

M12 showed a [M−H]^−^ ion at *m/z* 329.04741, which is 176 amu higher than the [M−H]^−^ ion of M11 (3,4-dihydroxybenzoic acid), indicating glucuronidation of M11. From the product scan spectrum of M12, characteristic product ions were observed at *m/z* 153.0184 (loss of the glucuronosyl moiety from the [M−H]^−^ ion), *m/z* 175.0240 (glucuronosyl moiety), and *m/z* 113.0233 (loss of CO_2_ and H_2_O from *m/z* 175.0240) (Figure 4). Incubation of 3,4-dihydroxybenzoic acid with rat liver S9 fraction in the presence of UDPGA resulted in M12. From these results, M12 was tentatively identified as M11 (3,4-dihydroxybenzoic acid) glucuronide but the position of glucuronidation was not accurately identified.

M13 and M14 were identified in the urine samples and after incubation of verproside with rat hepatocytes, and they each showed a [M−H]^−^ ion at *m/z* 232.97580, which is 80 amu higher than the [M−H]^−^ ion of M11 (3,4-dihydroxybenzoic acid), suggesting sulfation of M11. M13 and M14 were produced after incubation of 3,4-dihydroxybenzoic acid with rat liver S9 fraction in the presence of PAPS. M13 and M14 generated characteristic product ions at *m/z* 153.0184 (loss of SO_3_ from the [M−H]^−^ ion) and *m/z* 109.0283 (loss of CO_2_ from *m/z* 153.0184). M13 and M14 were tentatively characterized as M11 (3,4-dihydroxybenzoic acid) sulfates, but the exact site for sulfation could not be determined.

M15 and M16 each showed an [M−H]^−^ ion at *m/z* 167.03388 and generated characteristic product ions at *m/z* 152.0105 (loss of CH_3_ from the [M−H]^−^ ion), *m/z* 123.0440 (loss of CO_2_ from the [M−H]^−^ ion), and *m/z* 108.0204 (loss of CH_3_ and CO_2_ from the [M−H]^−^ ion) (Figure 4). By comparison with the retention times and the product scan spectra of the corresponding authentic standards, M15 and M16 were identified as 3-methoxy-4-hydroxybenzoic acid and 3-hydroxy-4-methoxybenzoic acid, respectively.

M17, M18, and M19 each showed a [M−H]^−^ ion at *m/z* 343.06759, which is 176 amu higher than the [M−H]^−^ ion of M15 (3-methoxy-4-hydroxybenzoic acid) and M16 (3-hydroxy-4-methoxy-benzoic acid), indicating glucuronidation of M15 and M16. M17, M18, and M19 generated characteristic product ions at *m/z* 167.0342 (loss of glucuronosyl moiety from the [M−H]^−^ ion), *m/z* 152.0105 (loss of CH_3_ from *m/z* 167.0342), *m/z* 175.0241 (glucuronosyl moiety) and *m/z* 113.0233. While M17 and M18 were formed from 3-methoxy-4-hydroxybenzoic acid (M15) after incubation of rat liver S9 in the presence of UDPGA, M19 was produced from incubation of 3-hydroxy-4-methoxybenzoic acid. From these results, M17 and M18 were tentatively identified as M15 (3-methoxy-4-hydroxybenzoic acid) glucuronides, and M19 was tentatively identified as M16 (3-hydroxy-4-methoxybenzoic acid) glucuronide. The position of glucuronidation was not accurately identified.

M20 and M21 each showed a [M−H]^−^ ion at *m/z* 246.99086, which was 80 amu higher than the [M−H]^−^ ion of M15 (3-methoxy-4-hydroxybenzoic acid) and M16 (3-hydroxy-4-methoxybenzoic acid), suggesting sulfation of M15 and M16. The product ion spectra of M20 and M21 generated the characteristic product ion at *m/z* 167.0343 (loss of SO_3_ from the [M−H]^−^ ion) (Figure 4). Incubation of 3-methoxy-4-hydroxybenzoic acid and 3-hydroxy-4-methoxybenzoic acid with rat liver S9 fraction in the presence of PAPS resulted in M20 and M21, respectively. M20 and M21 were tentatively identified as M15 (3-methoxy-4-hydroxybenzoic acid) sulfate and M16 (3-hydroxy-4-methoxybenzoic acid) sulfate, respectively.

**Scheme 1:**
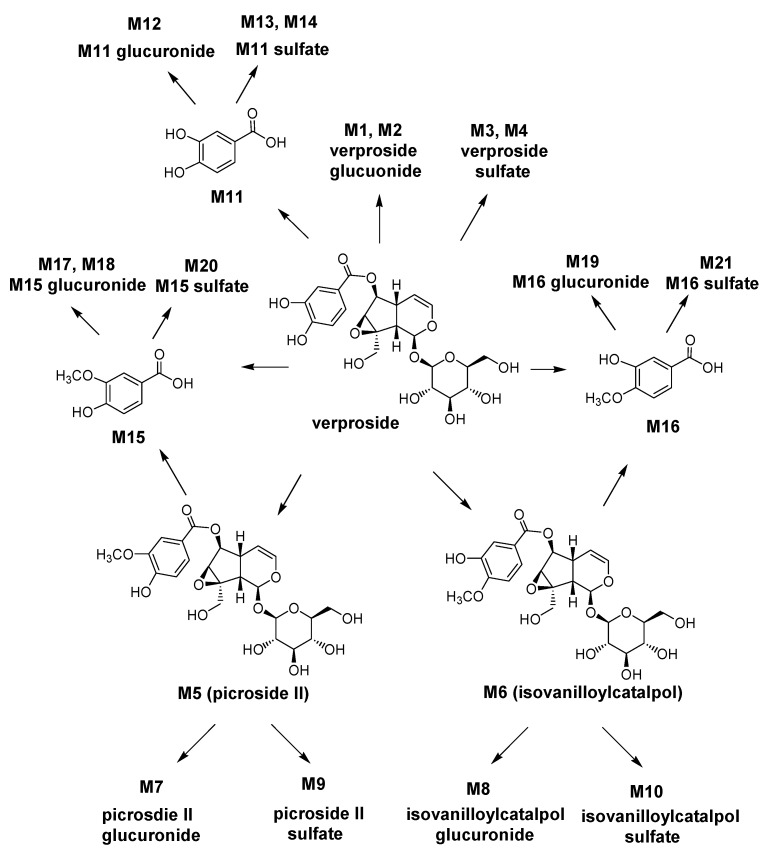
Possible metabolic pathways of verproside in rats.

On the basis of these results, the possible metabolic pathways of verproside in rats are shown in Scheme 1. Verproside is metabolized to verproside glucuronides (M1 and M2), verproside sulfates (M3 and M4), *O*-methylverproside such as picroside II (M5) and isovanilloylcatalpol (M6), 3,4-dihydroxybenzoic acid (M11), 3-methyoxy-4-hydroxybenzoic acid (M15) and 3-hydroxy-4-methoxybenzoic acid (M16), which are further metabolized to their glucuronides and sulfates including M5 glucuronide (M7), M5 sulfate (M9), M6 glucuronide (M8), M6 sulfate (M10), M11 glucuronide (M12), M11 sulfates (M13 and M14), M15 glucuronides (M17 and M18), M15 sulfate (M20), M16 glucuronide (M19), and M16 sulfate (M21). 

M1-M11, M13, M14, and verproside represented approximately 0.02, 0.33, 0.11, 10.82, 0.20, 0.01, 0.27, 0.20, 0.48, 0.12, 0.31, 0.09 (M13 and M14), and 87.05% of the total integrated peak areas, respectively, after incubation of verproside with rat hepatocytes for 2 hour, suggesting that verproside sulfation was a major metabolic pathway in rat hepatocytes. The percentages of the intravenous dose recovered as unchanged verproside were 0.77 (±0.59)% in 24 h bile sample and 4.48 (±2.47)% in 24 h urine sample, indicating that verproside may be extensively metabolized. The recovery of picroside II (M5) in 24 h bile and urine samples was 9.54 (±2.40)% and 5.10 (±1.45)%, respectively, but only a little isovanilloylcatalpol (M6) was recovered in urine and bile samples [less than 0.52 (±0.18)%]. After treatment of 24 h bile samples with b-glucuronidase, the biliary recoveries of verproside glucuronides (M1 and M2), M5 glucuronide (M7), and M6 glucuronide (M8) were *ca.* 0.44 (±0.19)%, 13.09 (±5.45)%, and 4.14 (±1.85)%, respectively. These results indicate that *O*-methylation of verproside to picroside II (M5) and isovanilloylcatalpol (M6) was the major metabolic pathway compared to glucuronidation and sulfation of verproside in rats.

## 3. Experimental

### 3.1. Materials

Verproside, isovanilloylcatalpol, and picroside II were isolated from *Pseudolysimachion rotundum *var*. subintegrum *(*Veronica spuria* var. *subintegra*), which was cultivated in Eumseong-gun, Korea and collected in September, 2009, using our previous method [6]. Their purities were determined to be more than 99.5% by HPLC. Nicotinamide adenine dinucleotide phosphate (reduced form) (NADPH), alamethicin, PAPS, UDPGA, b-glucuronidase (from *E. coli*), and sulfatase (from *Aerobacter aerogenes*) were obtained from Sigma-Aldrich Co. (St. Louis, MO, USA). Pooled male rat liver S9 fractions, cryopreserved pooled male rat hepatocytes, and cryopreserved hepatocyte purification kit were obtained from BD Bioscience Co. (San Jose, CA, USA). Methanol (HPLC grade) was obtained from Burdick & Jackson Inc. (SK Chemicals, Ulsan, Korea). Other chemicals were of the highest quality available. ProteoMass LTQ/FT-hybrid ESI Positive mode Cal Mix (MSCAL5) and Negative mode Cal Mix (MSCAL6) for the calibration of Q-Exactive MS were obtained from Supelco (Bellefonte, PA, USA).

### 3.2. *In Vivo* Metabolism of Verproside in the Rats

Four male Sprague-Dawley rats (230 ± 10 g, Samtako Co., Osan, Korea) were anaesthetized via intraperitoneal injection of zoletil (50 mg/kg) and the bile duct was catheterized using PE-10 tubing. The cannulated rat was kept in Ballman cage individually. Bile and urine samples were collected for 24 h after intravenous administration of verproside dissolved in sterile water to the tail vein at a dose of 10 mg/kg. Bile and urine samples were kept in −20 °C until analysis. The bile and urine samples (50 μL) were mixed with 50 μL of methanol and centrifuged. The supernatant (40 μL) was diluted with 160 μL of water. An aliquot (5 μL) was analyzed by the LC-HRMS method to identify verproside and its possible metabolites.

To characterize the nature of glucuronidation and sulfation, the bile and urine samples (25 μL) were incubated with b-glucuronidase (18.75 unit) or sulfatase (0.97 unit) at 37 °C for 1 h and were analyzed as described above. 

### 3.3. *In Vitro* Metabolism of Verproside and Its Possible Metabolites in Rat Hepatocytes and Liver S9 Fractions

Cryopreserved rat hepatocytes were recovered with a cryopreserved hepatocyte purification kit and viable hepatocytes were resuspended in Krebs-Henseleit buffer at a final concentration of 1.6 × 10^6^ cells/mL. 100 μL of rat hepatocyte suspensions (1.6 × 10^5^ cells) and 100 μL of 100 μM verproside were added into a 96 well plate and the mixture was incubated for 120 min at 37 °C in a CO_2_ incubator. 40 μL of the incubation mixture was transferred to a 1.5 mL eppendorf tube and mixed with 40 μL of methanol followed by centrifugation at 10,000 *×g* for 5 min. The supernatant (40 μL) was diluted with 60 μL of water and an aliquot (5 μL) was injected onto the LC-HRMS system.

The reaction mixture contained 50 mM potassium phosphate buffer (pH 7.4), 3 mM magnesium chloride, rat liver S9 fractions (200 μg protein), NADPH (1.3 mM), 3 mM UDPGA or 4 mM PAPS, and verproside (50 μM) in a total volume of 200 μL. To help the confirmation of the metabolites from verproside, authentic picroside II (50 μM), isovanilloylcatalpol (50 μM), 3,4-dihydroxybenzoic acid (200 μM), 3-methoxy-4-dihydroxybenzoic acid (200 μM), and 3-hydroxy-4-methoxybenzoic acid (200 μM) were also incubated as described above. Control incubations were conducted in the absence of NADPH, UDPGA, and PAPS. The samples were incubated at 37 °C for 60 min and the reaction was stopped by adding 400 μL of methanol. The reaction mixture was centrifuged and the supernatant was evaporated. The residue was dissolved in 100 μL of 15% methanol and an aliquot (5 μL) was injected onto the LC-HRMS system.

### 3.4. LC-HRMS Analysis of Verproside and Metabolites

To separate and identify the structures of verproside and its metabolites, a Q Exactive Orbitrap mass spectrometer (Thermo Scientific, San Jose, CA, USA) coupled with an Accela UPLC system was used. For separation of verproside and its metabolites, various columns such as Halo C18 column (2.7 μm, 2.1 mm i.d. × 50 mm, Advanced Materials Technology, Wilmington, DE, USA), Accucore MS C18 column (2.6 μm, 2.1 mm i.d. × 50 mm, Thermo Scientific), Luna phenylhexyl column (5.0 μm, 2.0 mm i.d. × 100 mm, Phenomenex, Torrance, CA, USA), Kinetex PFP column (2.6 μm, 2.1 mm i.d. × 50 mm, Phenomenex), and other various hydrophilic interaction chromatographic (HILIC) columns such as Cosmosil HILIC (5 μm, 2.0 mm i.d. × 100 mm, Nakalai Tesque Inc., Kyoto, Japan), Atlantis HILIC (3 μm, 3.0 mm i.d. × 50 mm, Waters, Milford, MA, USA), Kinetex HILIC (2.6 μm, 2.1 mm i.d. × 100 mm, Phenomenex), and SeQuant Zic HILIC (3.5 μm, 2.1 mm i.d. × 50 mm, Merck KGaA, Darmstadt, Germany) were tried using the gradient elution of methanol and ammonium formate buffer (1 mM, pH 3.1) as the mobile phase. Best separation of metabolites was obtained with a Halo C18 column using a gradient elution of 5% methanol in ammonium formate (1 mM, pH 3.1) (mobile phase A) and methanol (mobile phase B) at a flow rate of 0.5 mL/min: 5% mobile phase B for 3 min, 5% to 25% mobile phase B in 6 min, 25% to 90% mobile phase B in 0.4 min, 90% mobile phase B for 4.5 min, 90% to 13% mobile phase B in 0.4 min, 13% mobile phase B for 4 min. The column and the autosampler were maintained at 40 °C and 6 °C, respectively.

Accurate mass measurements for verproside and its metabolites were performed by electrospray ionization in the negative mode with the following electrospray source settings: ion transfer capillary temperature, 330 °C; needle spray voltage, −3,000 V; capillary voltage, −40 V; nitrogen sheath gas, 50 arbitrary unit; auxiliary gas, 15 arbitrary unit. Automatic gain control and resolution settings were scaled to 1,000,000 and 70,000, respectively. MS data were acquired using an external calibration in the scan range of *m/z* 100–700 and were processed using Xcalibur software version 2.2 (Thermo Scientific). Q Exactive Orbirap MS was calibrated with MSCAL5 and MSCAL6 for the positive ion mode and the negative ion mode, respectively, when daily results of mass accuracy deviation reached ± 5 ppm. Higher-energy collision dissociation (HCD) with nitrogen gas and collision energy of 25 eV were used in order to obtain product ion spectra of verproside and its metabolites. The proposed structures for the product ions of verproside and its metabolites were determined using the Mass Frontier software (version 6.0; HighChem Ltd., Bratislava, Slovakia).

## 4. Conclusions

The metabolic pathways of verproside in rats were elucidated for the first time in the present study. Verproside was metabolized to the following twenty-one metabolites in rats by means of *O*-methylation, glucuronidation, sulfation, and hydrolysis: verproside glucuronides (M1 and M2), verproside sulfates (M3 and M4), picroside II (M5), isovanilloylcatalpol (M6), M5 glucuronide (M7), M6 glucuronide (M8), M5 sulfate (M9), M6 sulfate (M10), 3,4-dihydroxybenzoic acid (M11), M11 glucuronide (M12), M11 sulfates (M13 and M14), 3-methoxy-4-hydroxybenzoic acid (M15), 3-hydroxy-4-methoxybenzoic acid (M16), M15 glucuronides (M17 and M18), M16 glucuronide (M19), M15 sulfate (M20), and M16 sulfate (M21). Incubation of verproside with rat hepatocytes resulted in formation of M1-M11, M13, M14, and verproside sulfate, M4 was identified as a major metabolite. After intravenous administration of verproside in rats, a little amount of verproside was recovered in bile (0.77% of the dose) and urine (4.48 of the dose), and *O*-methylation of verproside to picroside II (M5) and isovanilloylcatalpol (M6) followed by glucuronidation and sulfation was identified as the major metabolic pathway in bile and urine samples. 
